# Spreading Depolarizations Occur in Mild Traumatic Brain Injuries and Are Associated with Postinjury Behavior

**DOI:** 10.1523/ENEURO.0070-19.2019

**Published:** 2019-12-04

**Authors:** Johann M. Pacheco, Ashlyn Hines-Lanham, Claire Stratton, Carissa J. Mehos, Kathryn E. McCurdy, Natalie J. Pinkowski, Haikun Zhang, C. William Shuttleworth, Russell A. Morton

**Affiliations:** Department of Neurosciences, University of New Mexico School of Medicine, Albuquerque, NM 87131

**Keywords:** cerebral blood flow, closed skull impact, concussion, cortical spreading depression, electrophysiology, laser speckle contrast imaging

## Abstract

Millions of people suffer mild traumatic brain injuries (mTBIs) every year, and there is growing evidence that repeated injuries can result in long-term pathology. The acute symptoms of these injuries may or may not include the loss of consciousness but do include disorientation, confusion, and/or the inability to concentrate. Most of these acute symptoms spontaneously resolve within a few hours or days. However, the underlying physiological and cellular mechanisms remain unclear. Spreading depolarizations (SDs) are known to occur in rodents and humans following moderate and severe TBIs, and SDs have long been hypothesized to occur in more mild injuries. Using a closed skull impact model, we investigated the presence of SDs immediately following a mTBI. Animals remained motionless for multiple minutes following an impact and once recovered had fewer episodes of movement. We recorded the defining electrophysiological properties of SDs, including the large extracellular field potential shifts and suppression of high-frequency cortical activity. Impact-induced SDs were also associated with a propagating wave of reduced cerebral blood flow (CBF). In the wake of the SD, there was a prolonged period of reduced CBF that recovered in approximately 90 min. Similar to SDs in more severe injuries, the impact-induced SDs could be blocked with ketamine. Interestingly, impacts at a slower velocity did not produce the prolonged immobility and did not initiate SDs. Our data suggest that SDs play a significant role in mTBIs and SDs may contribute to the acute symptoms of mTBIs.

## Abstract (precis)

Millions of people suffer mild traumatic brain injuries (mTBIs) every year, and there is growing evidence that repeated injuries can result in long-term pathology. The acute symptoms of these injuries may or may not include the loss of consciousness but do include disorientation, confusion, and/or the inability to concentrate. Most of these acute symptoms spontaneously resolve within a few hours or days. However, the underlying physiological and cellular mechanisms remain unclear.

## Significance Statement

Millions of people suffer from concussions every year and repeated concussions are associated with chronic traumatic encephalopathy (CTE). Spreading depolarizations (SDs) are propagating waves of brain tissue depolarization that have been associated with strokes, subarachnoid hemorrhages, and moderate to severe brain injuries. SDs have long been hypothesized to occur in mild brain injuries, but have not been recorded. Our studies are the first to directly record the electrophysiological properties of SDs following a closed skull impact, and suggest that SDs may contribute to the acute symptoms of mild traumatic brain injuries (mTBIs).

## Introduction

Concussions are serious injuries and are now considered to be mild traumatic brain injuries (mTBIs). It is estimated that there are ∼3.8 million sports-related concussions each year (Clay et al., 2013). This is most likely an underestimate due to under reporting and/or a lack of a diagnosis. Concussions are often defined by the immediate and transient symptoms associated with impaired neurologic function. Most individuals recover within a few days or weeks, but ∼20–30% suffer from a constellation of prolonged symptoms including the inability to concentrate, dizziness, headaches, behavioral deficits, cognitive deficits, and/or sleep disruption for months (Silver et al., 2009; Brent and Max, 2017). In addition to cognitive and behavioral deficits, repeated mTBIs are associated with diffuse axonal injury, accumulation of amyloid precursor protein (APP), increases in amyloid-β, diffuse Aβ plaques, which collectively are used to diagnose chronic traumatic encephalopathy (CTE; for review, see DeKosky et al., 2013). A mTBI can disrupt cellular membranes, intracellular scaffolding, axonal fibers, synaptic connections, cerebral blood flow (CBF), and the blood brain barrier (Chodobski et al., 2011; Giza and Hovda, 2014). mTBIs are most commonly associated with reduced CBF (McLaughlin and Marion, 1996; Dietrich et al., 2000; Giri et al., 2000) and neurochemical and ion imbalances (Denny-Brown and Russell, 1941; Ward, 1964; Takahashi et al., 1981; West et al., 1982; Kubota et al., 1989; Katayama et al., 1990; Yoshino et al., 1991). Recovery from this imbalance is metabolically demanding and is likely to occur in the presence of reduced blood flow. This imbalance has been described as a neurometabolic cascade and can take days to weeks to fully recover (Giza and Hovda, 2014). It has been suggested that the underlying component of the neurometabolic cascade could be spreading depolarizations (SDs), but the role of SDs in mild injuries remains unclear.

SDs are propagating waves of complete tissue depolarization that result in a transient suppression of cortical activity lasting multiple minutes. SDs were first characterized in rabbits (Leao, 1944) and later extensively studied in rodents (Buresova, 1956; Bures and Buresova, 1960; Fifkova et al., 1961; Krivanek, 1961; Monakhov et al., 1962; Ruediger et al., 1962; Ward and Sinnett, 1971; Maxson and Cowen, 1977). Over 10 years of clinical recordings have implicated SDs in humans following traumatic brain injuries and strokes (Strong et al., 2002, 2007; Hartings et al., 2008, 2011). SDs have also been linked to the visual auras of migraine sufferers (Lauritzen et al., 1983; Cao et al., 1999; Hadjikhani et al., 2001). It has long been thought that SDs occur during mTBIs due to the similarities in the neurochemical imbalance and disruptions in CBF (Meyer and Denny-Brown, 1955; Zachar and Zacharová, 1958, 1961; Kubota et al., 1989; West et al., 1982; Watanabe and Noriaki, 2002). However, exciting new data from Bouley and colleagues described the hemodynamic responses that are associated with SDs immediately following an injury (Bouley et al., 2019). The hemodynamic response immediately following the injury was followed by a period of prolonged post-SD oligemia and was associated with neurologic dysfunction hours after the injury (Bouley et al., 2019). The electrophysiological properties of SDs have been recorded in more invasive models of mTBIs (i.e., fluid percussion and cortical impact; Takahashi et al., 1981; Kubota et al., 1989; Sunami and Nakamura, 1989; Nilsson et al., 1993; Sword et al., 2013). However, direct electrophysiological recordings of SDs have not been established in a mild closed skull injury model.

In this study, we use a mouse model of mTBI that produces concussion-like behavior, but does not result in gross tissue damage. SDs are defined by their electrophysiological properties of a large extracellular field potential shift and suppression of high-frequency cortical activity. We have directly recorded these electrical events in our model, and have correlated the presence of SDs to the behavioral phenotype of our model.

## Materials and Methods

### Animals

All animal procedures were performed in accordance with the authors’ University Institutional Animal Care and Use Committee. Wild-type C57/Bl6 mice were purchased from The Jackson Laboratory. To model late adolescence and early adulthood we used mice between eight and 14 weeks of age. Both male and female mice were used for these studies and were analyzed separately to identify any sex related differences before combining. Our preliminary analyses did not indicate any differences between the sexes, so all of our data sets include both male and female mice in equivalent numbers.

### mTBI model

**Figure 1.**
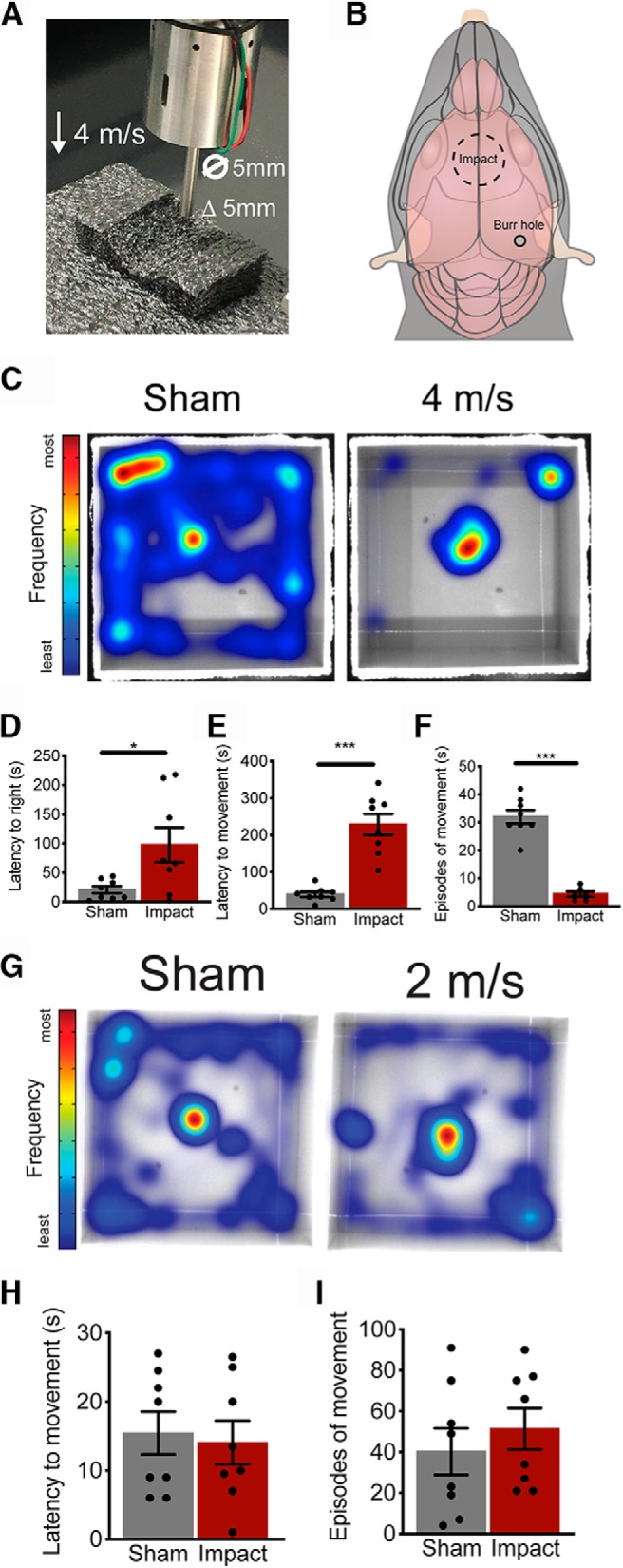
Closed skull impacts induce mTBI-like behavior. ***A***, Animals are anesthetized with isoflurane and placed on a custom Kaizen foam platform and impacted without restricting the head. Animals were impacted on top of the head at 2 or 4 m/s with a 5-mm diameter and a 5-mm deflection. ***B***, Schematic representation of the impact site including the position of the burr hole for the electrophysiological recordings. ***C***, Representative heatmaps indicating positions most frequently visited in warmer colors. The impacted animals displayed a prolonged time in the center following the impact and did not explore the arena as the sham-treated animals during the entire 10-min trial. Cumulative data for the latency to right themselves (***D***), latency to regain movement (***E***), and total episodes of movements (***F***) over the entire 10-min trial. Representative heatmaps for sham and 2-m/s impacted animals show similar time in the center and exploratory behavior (***G***). There was no significant difference in the latency to regain movement (***H***) and total episodes of movement (***I***) between sham and 2-m/s animals.

The model used in these studies was adapted from previously published work using controlled cortical impactors without craniotomy (Lighthall, 1988; Shitaka et al., 2011; Main et al., 2017). The modification here is that the motion of the head and body was not restricted during the impact to allow for coup and countercoup motion. Animals were anesthetized with either isoflurane (2–4%) or urethane (1.5 mg/g). The anesthetized animal was placed on a custom Kaizen foam (FastCap) platform directly beneath the impactor without restricting the body or head. The Impact One controlled impactor (Leica Biosystems) was used to deliver the impact to the intact skin and skull. The foam platform and impactor are shown in Figure 1*A*. The animal’s head was not restrained during the impact. The 5 mm impactor tip was positioned with the front edge of the impact tip aligned with the eyes resulting in the impact center at ∼1 mm rostral to bregma. A schematic representation of the impact site is show in Figure 1*B*. The impactor tip was lowered to press the animals head down to the surface of the platform before tip retraction. The impactor was then lowered an additional 5 mm for the deflection. The animals were impacted at a speed of 4 or 2 m/s with a dwell time of 20 ms. The impact resulted in the deflection of the animal’s head away from the impact site. Sham-treated animals were anesthetized, placed on the platform, and the impactor tip was lowered to touch the animal’s head.

### Behavior of mTBI

Mice were anesthetized with 2–4% isoflurane (Clipper Distribution Company) supplemented with oxygen at a flow rate of 1 L/min. Isoflurane anesthesia allowed rapid recovery from anesthesia to monitor short-term behavioral effects. Following an impact, the animals were immediately placed on their side in the center of an open field arena. This permitted tracking of recovery and behavior within seconds of the impact. Behavior was monitored for 10 min using the Ethovison motion tracking system (Noldus). The time to righting reflex and initial movement was manually assessed. Recovery of movement was considered to be the first full step of the animal. Total distance traveled and episodes of movement were automatically calculated by the Ethovision system. The parameters were set so that velocities above 4.5 m/s were considered moving and velocities below 1.75 m/s was considered not moving. Intermediate velocities were excluded from the analysis. Movement heatmaps were generated for each animal for a 10-min trial.

### Behavioral and cognitive tasks

#### Home cage monitoring

We used the photobeam activity system (PAS) (San Diego Instruments) to monitor behavior continuously for multiple days after the impact. Animals were singly housed with 1/4-inch corncob bedding ∼3/8 of an inch deep under a normal light cycle. Animals were acclimated to the corncob bedding for 24 h before monitoring. Each cage was placed within the photobeam detectors and beam breaks were accumulated into 5-min bins. Animals were monitored for 24 h before treatment. Animals were only removed from their cage for anesthesia and treatment (sham vs mTBI), and immediately placed back into their cage within 5 s of the impact. Animals were monitored for an additional 5 d. Acute mobility was assessed for 4 h following treatment (5-min bins) and long-tern mobility was assessed for 7 d, beginning 24 h before treatment (60-min bins).

#### Open field and novel object recognition

Open field behavior was monitored via the Ethovison motion tracking system 24 h after the impact to broadly assess mobility and anxiety. The arena was well lit with ∼300 lux in the center of the arena and ∼200 lux in the corners. Each animal was placed into the center of the arena and monitored for 10 min before returning back to its home cage. Total times spent in the center and border regions were calculated, as well as the total distance traveled. The novel object task was used to assess learning and memory 48 h after the impact. The open field setup was used with identical settings and two identical objects were placed at the opposite corners of the arena. The animals were allowed to explore the objects for 5 min. The animals were removed from the arena and the two objects and the arena was wiped down. One identical object and one novel object were placed back into the arena. Within 5 min, the mice were allowed to explore the two objects for 5 min. The total duration of time spent investigating the identical and novel objects were quantified as a measure of learning and memory.

#### CatWalk gait analysis

We used the Noldus CatWalk system to quantify gait characteristics 72 h after the impact. Individual animals were placed on the glass platform and the animals were allowed to freely walk up and down the CatWalk. A camera from below captured the animal’s footprint for automated analysis. The following parameters were used for a successful run: minimum duration of 0.5 s, maximum duration of 10 s, and during each run the animal’s speed could not change >65%. Each run was classified and the following characteristics were analyzed to assess for gait abnormalities: print area, swing duration, and stride length. Print area was calculated by averaging the area of all the footprints for each individual paw. Swing duration was considered to be the time between the lifting and replacing of the same paw. The stride length was calculated by the distance from the heel of one footprint to the heel of the next footprint of the same paw.

#### Trace fear conditioning

We used fear conditioning boxes that were equipped with infrared motion tracking capabilities (Med Associates Inc). Animals were treated 24 h before fear conditioning. Animals were conditioned to the box 120 s before the first 30-s tone that was followed by a 20-s delay and a 2-s foot shock at 0.6 mA. This was repeated five times with an average inter-event interval of 120 s. On day 2, the fear conditioning boxes were moved to a different location, the walls and floors were change, and a vanilla scent was added to the behavior box. Animals were again placed into the box, given the tone for 30 s with no foot shock. This was repeated three times with an inter-event interval of 220 s. On day 3, the behavior boxes were moved back to the previous location with the original walls and floors. The animals were place into the box for a total of 480 s. The automated motion tracking capabilities of the behavior boxes was used to assess freezing and the percentage time freezing was calculated. Freezing was calculated for day 2 during the tone and delay period of 220-s delay. For day 3, the entire 480 s was assessed for freezing behavior.

### Laser speckle contrast imaging (LSCI) and electrophysiological recordings

Animals were anesthetized with 1.5 mg/g urethane (Sigma-Aldrich) before treatment and remained under anesthesia for the entirety of the experiment. Following the completion of the experiment the animals were sacrificed by decapitation. LSCI (Boas and Dunn, 2010; Miao et al., 2011) was used to monitor CBF through the intact skull. A skin flap was created to expose the skull and the skull was illuminated with a 785 nm laser diode (Thorlabs). The scattered light was collected through a 720 nm long pass filter and a Nikon 18- to 55-mm manual focus lens (f-stop between 3.5 and 5.6) attached to a stingray F-504 CCD camera (Allied Vision). Blood flow maps were calculated and displayed using LabView software (National Instruments) modified from Choi and colleagues (Yang et al., 2011). Normalized blood flow measurements were calculated from the LSCI images using ImageJ (NIH). Blood flow was quantified by creating a 600 × 600 μm region of interest (ROI) over the visual cortex in either hemisphere. The averaged pixel intensity of that region was calculated and plotted over time to demonstrate the dynamic blood flow changes that are associated with SDs. For the reperfusion data set, 100 individual images were averaged together to represent each time point and a 600 × 600 μm ROI was again used over the visual cortex or major vessel. ROI placement was based on a consistent anatomic location throughout the repeated measurements. All data were normalized to pre-impact LSCI levels. Chronic windows were created to monitor CBF for multiple days following a single impact. An incision was generated over the right hemisphere above the visual cortex and the underlying skull was exposed and cleaned. A drop of cyanoacrylate glue was placed on the skull and a 4 mm diameter cover glass was placed on the glue and skull. The skin was securely glued to the underlying skull and cover glass. Animals were allowed to recover from the surgery for 24–48 h before treatment.

Electrophysiological recordings were done with an Axon 2B amplifier (Molecular Devices) equipped with a HS-2A headstage. Data were acquired at 10 kHz using a PowerLab 8/35 (AD Instruments) acquisition system and LabChart 7. Glass electrodes were pulled with a P-97 Flaming/Brown Micropipette puller (Sutter Instruments) and filled with artificial CSF (ACSF) containing the following: 125 mM NaCl, 2 mM KCl, 1.3 mM NaH_2_PO_4_, 26 mM NaCO_3_, 10 mM glucose, 2 mM CaCl_2_, and 1 mM MgSO_4_. Electrodes were placed ∼500 μm beneath the surface of the brain through a burr hole in the skull located over the visual cortex (Fig. 1*B*). The burr hole was generated 60 min before baseline recordings. To maintain the validity of the model, these recordings were done without head restriction resulting in some movement artifacts from animal respiration. Baseline electrophysiological data were acquired before the impact. The electrode was removed for the impact and then replaced within 5 s. These recordings were done with simultaneous LSCI to ensure that SD was not generated from the insertion of the electrode. Electrophysiological recordings were quantified following the recommendations of the Co-Operative Study on Brain Injury Depolarizations (COSBID) group (Dreier et al., 2017). In brief, the slow extracellular potential shifts of SD were assessed after the low pass filter was set at 5 Hz. SD onset was considered to occur at the initial drop of the DC shift and recovery was considered to be when the extracellular potential reached 85% of the pre-SD level. High-frequency activity was monitored using a bandpass filter between 0.5 and 45 Hz. Recovery of high-frequency activity was assessed from the total power of the high-frequency activity (V^2^). As recommended, the first spike in the total power signal was considered recovery of the high-frequency activity.

Ketamine was used as a pharmacological blocker of SDs to confirm that the impact-induced SDs were sensitive to NMDA receptor antagonists. Ketamine was administered via an intraperitoneal injection 30–45 min before the impact at a dose of 120 mg/kg (Hernándéz-Cáceres et al., 1987; von Bornstädt et al., 2015). Due to the anesthetic actions of ketamine at this concentration, the urethane dosage was halved and the animals were monitored for a pain response. Ketamine was given 10 min before the urethane and the impact was given within 45 min of the ketamine injection.

### Histology and immunohistochemistry

For histologic analysis the animals were sacrificed 24 h post-treatment. Animals were heavily anesthetized with isoflurane and were cardiac perfused first with ice-cold PBS containing 1 unit/mL heparin followed by ice-cold 4% paraformaldehyde (Sigma-Aldrich). Brains were removed and immediately submerged in 4% paraformaldehyde for 48 h at 4°C. Brains were sectioned using a vibrating vibratome (VT1000S, Leica) at a thickness of 50 μm. Sections were immediately placed in cryoprotection solution and maintained at –20°C.

For Nissl staining, sections were transferred to frosted slides and allowed to dry. Sections were then dehydrated with ethanol (2 × 3 min) and cleared with xylene (15 min). Sections were washed in 100% ethanol (2 × 3 min) and rinsed in tap water. Sections were stained for 4–8 min in 0.1% cresyl violet and rinsed with tap water then washed with 100% ethanol (2 × 3 min) before mounting in Permount (catalog #SP15, Fisher Chemical).

To assess for micro-bleeds or blood brain barrier disruptions we used a Prussian blue kit from Abcam (catalog #ab150674). In brief, sections were mounted on charged slides and allow to fully dry. Sections were rehydrated in PBS two times for 5 min each. Slides were dipped into diH_2_0 and placed into the iron stain for 5 min. Slides were immediately transferred to a NucRed stain for 2 min. Slides were washed four times in diH_2_0 and dehydrated with 95% and 100% ethanol for 2 min each. Sections were cleared in xylene for 5 min and mounted with Permount.

The *in situ* apoptosis detection kit from Sigma-Aldrich (catalog #S7100) was used to assess for cell death with TUNEL staining. Sections were mounted on charged slides and allowed to dry completely. Sections were rehydrated in PBS and the procedures from the kit were followed. DAB labeling was done with the ImmPACT DAB kit from Vector Laboratories (catalog #SK-4105). Images were converted to 8-bit gray scale and converted to red for the representative images in ImageJ.

For immunohistochemical assessment, sections were washed in PBS-T and then blocked using 5% normal donkey serum and 1% bovine serum albumin in PBS-T for 1 h. To assess for cell death, sections were stained with Fluoro-Jade C solution (0.0001% Fluoro-Jade C in 0.1% acetic acid; catalog #AG325, EMD Millipore Corp, Merk) for 10 min. Sections were washed with PBS-T twice for 10 min each and mounted. Glial fibrillary acidic protein (GFAP) was used as a marker of astrocyte activation and the primary antibody (catalog #NE1015, EMD Millipore, Merk) was diluted 1:500 in the blocking buffer and incubated at 4°C overnight. The following day, the sections were washed in PBS-T three times for 10 min each before being incubated with the secondary antibody [Cy3-IgG (H + L) donkey anti-mouse, catalog #715-167-003, Jackson ImmunoResearch] at room temperature for 2 h. Sections were washed three times for 10 min each in PBS-T, then mounted onto slides. All images were acquired with an Olympus IX71 inverted microscope equipped with an Olympus DP72 RGB camera through either a 4× UPlanFL N 0.13 na objective, 10× UPlanFl 0.3 na objective, or 40× LUCPlanFLN 0.60 na objective. Images were acquired and stitched with the Olympus cellSens software system and quantified with ImageJ.

### Statistical analysis

All statistical analyses were performed in Prism (v.8). All datasets were tested for a normal distribution using the D’Agostino–Pearson test, and for outliers using the Rout test with Q = 1%. All statistical analyses of pooled data were performed using a two-tailed Student’s *t* test or Mann–Whitney tests, and the level of significance was considered to be *p* < 0.05. All statistics are provided in the figure legends and data are presented as group means and SEMs. For details of results of statistical analyses, see Table 1.

**Table 1.** Statistics table

Description	Figure	Normal distribution	Method	Significant	*t* or *F*	*p* value
4-m/s latency to right	1*D*	Yes	D’Agostino–Pearson and S-W	Yes	*t* = 2.530	*p* = 0.024
4-m/s latency to movement	1*E*	Yes	D’Agostino–Pearson and S-W	Yes	*t* = 6.413	*p* < 0.0001
4-m/s episodes of movement	1*F*	Yes	D’Agostino–Pearson and S-W	Yes	*t* = 9.46	*p* < 0.0001
2-m/s latency to movement	1*H*	Yes	D’Agostino–Pearson and S-W	No	*t* = 0.309	*p* = 0.7618
2-m/s episodes of movement	1*I*	Yes	D’Agostino–Pearson and S-W	No	*t* = 0.7336	*p* = 0.4753
4-h home cage monitoring	2*A*	No	D’Agostino–Pearson and S-W	No	*F* = 0.0806	*p* = 0.781
5-d home cage monitoring	2*B*	No	D’Agostino–Pearson and S-W	No	*F* = 0.7883	*p* = 0.3747
24-h open field total dis	2*C*	Yes	D’Agostino–Pearson and S-W	No	*t* = 1.051	*p* = 0.311
24-h open field center	2*D*	No	D’Agostino–Pearson and S-W	No	M-W	*p* = 0.2345
24-h open field borders	2*E*	No	D’Agostino–Pearson and S-W	No	M-W	*p* = 0.2786
48-h NOR	2*F*	Yes	D’Agostino–Pearson and S-W	Yes	*F* = 5.741	*p* = 0.0235
72-h CatWalk print area RF	2*G*	Yes	D’Agostino–Pearson and S-W	No	*t* = 0.8259	*p* = 0.4284
72-h CatWalk print area RH	2*G*	Yes	D’Agostino–Pearson and S-W	No	*t* = 0.552	*p* = 0.5897
72-h CatWalk print area LF	2*G*	Yes	D’Agostino–Pearson and S-W	No	*t* = 0.0714	*p* = 0.9441
72-h CatWalk print area LH	2*G*	Yes	D’Agostino–Pearson and S-W	No	*t* = 0.3652	*p* = 0.7204
72-h CatWalk swing duration RF	2*H*	Yes	D’Agostino–Pearson and S-W	No	*t* = 0.5281	*p* = 0.5950
72-h CatWalk swing duration RH	2*H*	No	D’Agostino–Pearson and S-W	Yes	M-W	*p* = 0.0379
72-h CatWalk swing duration LF	2*H*	Yes	D’Agostino–Pearson and S-W	No	*t* = 1.228	*p* = 0.2398
72-h CatWalk swing duration LH	2*H*	Yes	S-W only	No	*t* = 0.1183	*p* = 0.9076
72-h CatWalk stride length RF	2*I*	Yes	D’Agostino–Pearson and S-W	No	*t* = 0.9193	*p* = 0.3735
72-h CatWalk stride length RH	2*I*	Yes	D’Agostino–Pearson and S-W	No	*t* = 1.249	*p* = 0.2321
72-h CatWalk stride length LF	2*I*	Yes	D’Agostino–Pearson and S-W	No	*t* = 0.7567	*p* = 0.4618
72-h CatWalk stride length LH	2*I*	Yes	D’Agostino–Pearson and S-W	No	*t* = 0.7110	*p* = 0.8666
GFAP quantification	3*E*	Yes	D’Agostino–Pearson only	No	*t* = 1.295	*p* = 0.2138
FluroJade quantification	3*F*	Yes	D’Agostino–Pearson and S-W	No	*t* = 0.8128	*p* = 0.4282
Prolonged CBF recovery tissue	5*B* overall	Yes	D’Agostino–Pearson and S-W			
	5*B* 0 min			No		*p* > 0.9999
	5*B* 5 min			No		*p* > 0.9999
	5*B* 15 min			Yes		*p* = 0.0033
	5*B* 30 min			Yes		*p* = 0028
	5*B* 45 min			Yes		*p* = 0.0001
	5*B* 60 min			Yes		*p* = 0.0014
	5*B* 75 min			No		*p* = 0.2927
	5*B* 90 min			No		*p* > 0.9999
	5*B* 105 min			No		*p* > 0.9999
	5*B* 120 min			No		*p* > 0.9999
Prolonged CBF recovery venous	5*C* overall	Yes	D’Agostino–Pearson and S-W	No		*p* > 0.9999
	5*C* 0 min			No		*p* > 0.9999
	5*C* 5 min			Yes		*p* = 0.0272
	5*C* 15 min			Yes		*p* = 0.0016
	5*C* 30 min			Yes		*p* = 0.0103
	5*C* 45 min			Yes		*p* = 0010
	5*C* 60 min			Yes		*p* = 0.0302
	5*C* 75 min			No		*p* = 0.6456
	5*C* 90 min			No		*p* > 0.9999
	5*C* 105 min			No		*p* > 0.9999
	5*C* 120 min			No		*p* > 0.9999
Multi-day CBF recovery	5*F*	No	S-W only			
						*p* > 0.9999
						*p* < 0.0001
						*p* = 0.5034
						*p* = 0.4908
						*p* = 0.9819
SD occurance Ure vs Iso	5*G*	Yes	D’Agostino–Pearson and S-W	No	*t* = 0.00902	*p* = 0.9929

## Results

### Closed skull impact results in acute mTBI-like behavior

We used video monitoring to investigate post-mTBI behavior. Animals were anesthetized with isoflurane and placed on a custom-made foam platform. The platform supported the head in a horizontal position, but did not restrict the head or body (Fig. 1*A*). The platform and impactor are shown in Figure 1*A*. A schematic representation of the impact site is shown in Figure 1*B*. Immediately following treatment, animals were placed in the center of an open field arena and monitored for 10 min. On average the mTBI animals righted themselves in 97.5 s compared to 20.6 s for the sham-treated animals (*t*_(14)_ = 2.53, *p* = 0.02; sham *n* = 8; mTBI *n* = 9; Fig. 1*D*). Representative heat maps are shown in Figure 1*C* to demonstrate the overall difference between the treatment groups. In addition to the righting reflex, the mTBI animals took on average 228.7 s to regain movement versus 39.1 s for the sham animals (*t*_(14)_ = 6.413, *p* < 0.0001; Fig. 1*E*). Following the recovery of movement, the mTBI animals averaged 4.3 episodes of movement whereas the sham-treated animals averaged 32 episodes (*t*_(12)_ = 9.46, *p* < 0.0001; Fig. 1*F*). This behavior resembled the acute disorientation symptoms of human mTBI, and led us to ask if this behavior was dependent on the severity of the impact. In a separate cohort, animals were impacted at 2 m/s instead of 4 m/s with all the other parameters maintained. Animals that were impacted at 2 m/s did not display the initial period of immobility (sham = 15.4 s vs 2 m/s = 14.06 s; *t*_(14)_ = 0.31, *p* = 0.762; Fig. 1*H*) and had similar episodes of movement (sham = 40.2 vs 2 m/s = 51.3; *t*_(14)_ = 0.734, *p* = 0.475; sham *n* = 8; 2 m/s impact *n* = 8; Fig. 1*I*). Representative heat maps indicate similar patterns movement between groups in the acute behavior following a 2-m/s impact (Fig. 1*G*).

### A single mTBI does not result in significant deficits in long-term behavior, gait, or learning and memory

**Figure 2.**
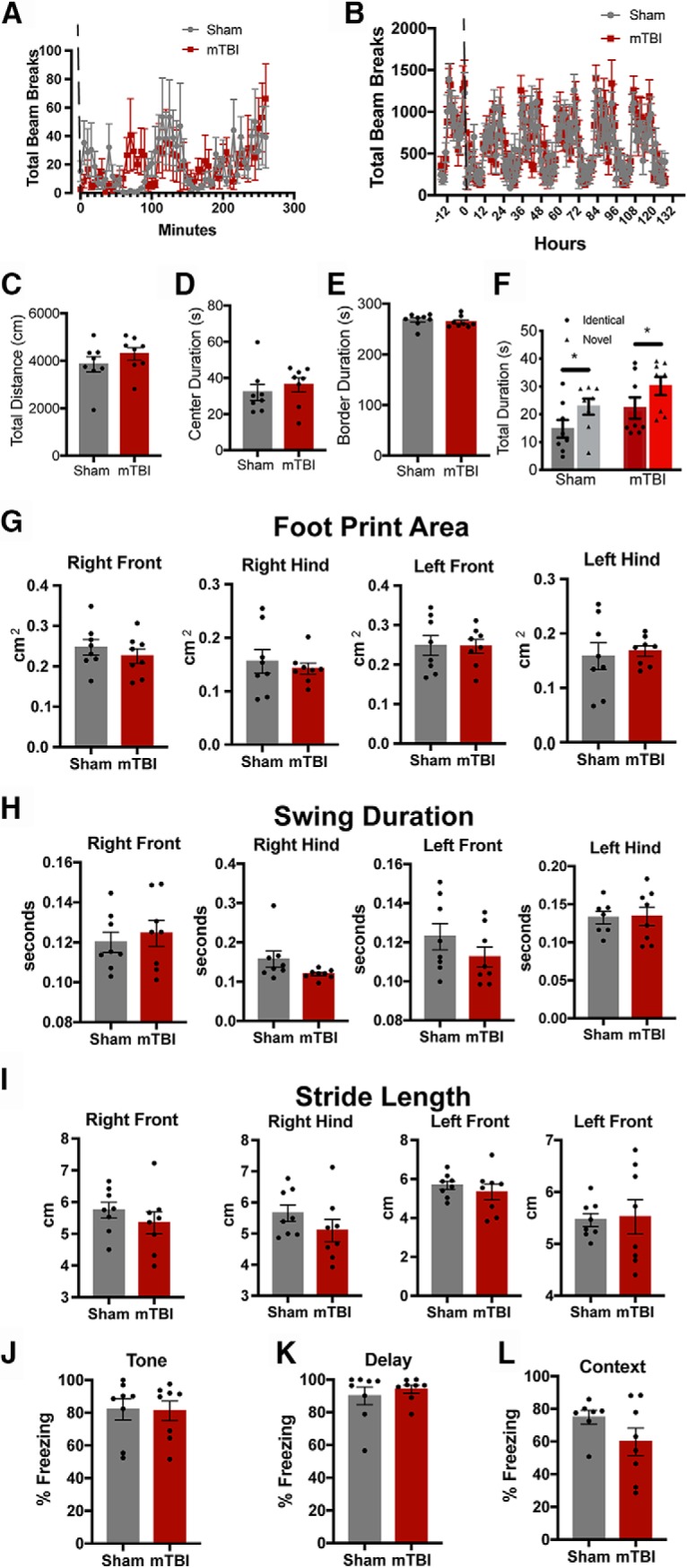
mTBIs do not produce long-term deficits in ambulatory activity, gait, or learning and memory. Ambulatory activity was assessed with a photobeam home cage monitoring system immediately following the 4-m/s impact for 5 d continuously. Activity was assessed for 4 h immediately after the treatment in 5-min bins (***A***). To assess sleep wake cycles, the activity was monitored 24 h before treatment and continuously for 6 d in 1-h bins (***B***). The dashed line indicates the time of treatment (sham vs impact). Open field behavior was assessed 24 h post-sham or mTBI (4 m/s) treatment to test for overall activity and anxiety. There was no significant difference in total distance traveled (***C***), time spent in the center (***D***), and time spent in the border regions (***E***). Novel object recognition was used to test for short-term learning and memory 48 h post-treatment. Animals were placed into the open field arena containing two objects and allowed to explore the objects for 5 min. Animals were removed and the arena, objects were cleaned, one identical object and one novel object were placed back into the arena, and the same animals was allowed to explore the objects for another 5 min. The time spent with each object was quantified (***F***). Using the Noldus CatWalk gait analysis system we quantified the footprint area (***G***), swing duration (***H***), and stride length (***I***) for each individual foot 72 h post-treatment. Contextual trace fear conditioning was also used to assess for hippocampal dysfunction. 24 h post-treatment animals were placed within the behavioral box and given a 30-s tone followed by a 20-s delay and a 0.6-mA foot shock for 2 s. This was repeated five times to learn the association. On day 2, the animals were put into a modified chamber and given the 30-s tone without the foot shock. Freezing was assessed during the 30-s tone (***J***) and the 20-s delay (***K***). On day 3, the animals were placed into the original context for 8 min. The percentage of the time freezing during that 8-min trial was quantified (***L***).

To assess behavior on a longer time scale we used a home cage photobeam system to monitor animal movement continuously for multiple hours and days. We were able to confirm our previous open field studies, by showing that the mTBI group had on average 2.5 beam breaks in the first 5 min compared to 15.2 in the sham treated (Fig. 2*A*). The mTBI animals were more active 1 h after the impact relative to the sham-treated animals, but *post hoc* analysis did not show any statistically significantly difference between the groups (*F*_(1,14)_ = 0.081, *p* = 0.781; Fig. 2*A*; Table 1). Furthermore, the active and sleep cycles were nearly identical in both treatment groups for five consecutive days (*F*_(1,2016)_ = 0.788, *p* = 0.375; Fig. 2*B*; Table 1). In a separate cohort of animals, we tested for activity and general anxiety using the open field task 24 h post-treatment. There was no statistical difference between the sham or mTBI animals in total distance traveled (*t*_(14)_ = 1.051, *p* = 0.311), in the duration spent in either the center (*t*_(14)_ = 0.704, *p* = 0.493), or duration in the border regions (*t*_(14)_ = 0.704, *p* = 0.493; Fig. 2*C–E*). To test for learning and memory we used the novel object recognition task 48 h post-treatment. Both treatment groups investigated the novel object significantly more than the familiar object (*F*_(1,28)_ = 5.741, *p* = 0.023) and there was no statistical difference between treatment groups (*F*_(1,28)_ = 0.0001, *p* = 0.991; Fig. 2*F*). One common deficit among mTBI models is the presence of gait abnormalities. To test for gait abnormalities 72 h post-treatment, we used the CatWalk automated system. We investigated the footprint area, swing duration, and stride length (Fig. 2*G–I*), which have been previously been shown to have deficits in a similar mTBI model (Mountney et al., 2017; Neumann et al., 2009). We were unable to detect any significant differences between the sham and mTBI animals (Table 1). In another cohort, we used the contextual fear-based task as a more sensitive measure for learning and memory. Animals were conditioned to the tone 24 h post-mTBI and then tested 24 h post-conditioning for freezing behavior in a different context. Animals were placed in a modified cage and given the associated tone with no foot shock. Both treatment groups had equivalent freezing during the tone (*t*_(14)_ = 0.095, *p* = 0.925; Fig. 2*G*) and the delay period (*t*_(14)_ = 0.688, *p* = 0.502; Fig. 2*H*). Twenty-four hours later, the animals were placed in the original context and total freezing was assessed. There was no statistical difference between the treatment groups in the total degree of freezing (*t*_(14)_ = 1.523, *p* = 0.151; Fig. 2*I*). Together, these data suggest that the mTBIs do not result in long-term deficits in mobility, sleep wake cycles, gait, or learning and memory.

### mTBI model does not produce significant tissue damage or astrocyte activation

**Figure 3.**
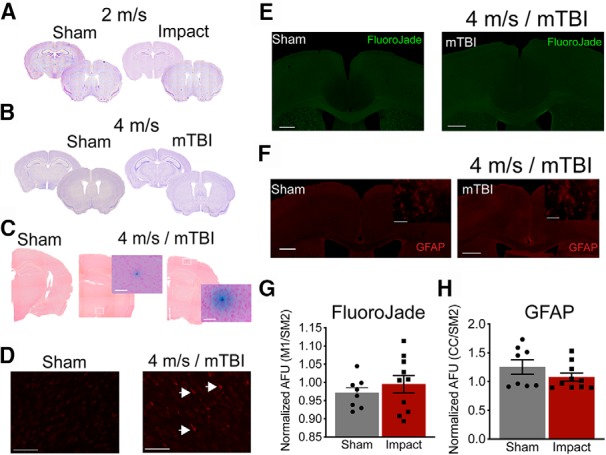
Impacts at 4 m/s (mTBI) do not produce significant tissue damage or astrocyte activation. Representative Nissl stains indicating no gross structural damage 24 h post-impact for 2 m/s (***A***) or 4 m/s (***B***). Prussian blue stain was used to identify microbleeds and the tissue was counter stained with NucRed. Three to four sections per animal were stained, and we only identified the two microbleeds shown in ***C*** from two separate mTBI animals. No other microbleeds were present in the sham or the other six mTBI animals. Representative images for sham-treated animals and the two microbleed sections taken at 4× and 40× magnification images of the microbleed themselves (***C***). The TUNEL stain was used to identify cells undergoing programed cell death. We only identified one mTBI animal that had detectable staining. Representative images are shown for sham-treated animals and the one mTBI animal that had TUNEL-positive cells shown by the arrow heads (***D***). Cell death was also assessed using FluoroJade-C. We were unable to detect a difference in overall fluorescent or individual cell bodies with positive staining. Representative images are shown (***E***). Neuroinflammation was assessed by GFAP staining. Representative images are shown (***F***). Again, we were unable to detect a difference in the overall fluorescence between the sham or mTBI (4 m/s) animals. Quantified fluorescence was measured by averaging the pixel intensity of the dorsal motor cortex to the lateral somatosensory cortex for sham and mTBI animals for FluoroJade (***G***) and GFAP staining (***H***). Scale bars = 500 μm (for the overview images) and 50 μm (for the increased magnification).

**Figure 4.**
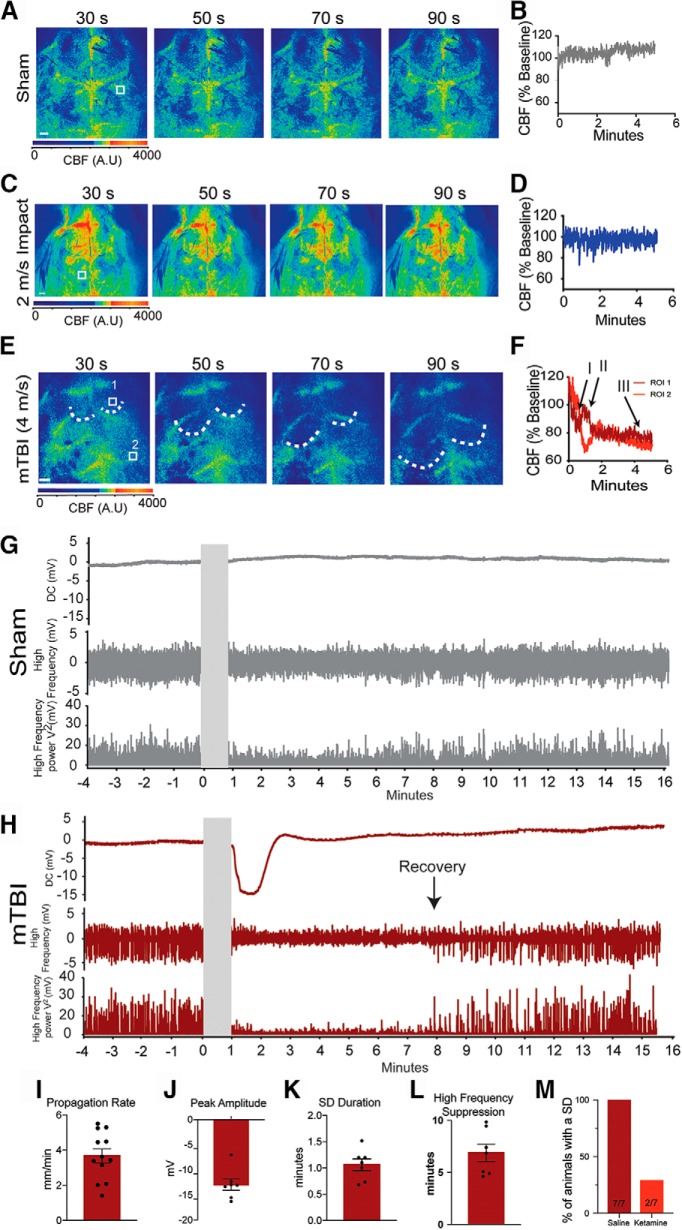
Impacts associated with mTBI-like behavior produce SD. LSCI was used to assess CBF before and immediately after treatment. Representative LSCI images from a sham (***A***), 2 m/s (***C***), and mTBI (4 m/s; ***E***) animals are shown. Warmer colors indicate more blood flow and the white boxes indicate the ROIs used to create the time plots. Scale bars = 500 μm (in the LSCI images). Dotted lines indicate the leading edge of the propagation wave. Graphical representations of the CBF from the indicated ROIs are shown for sham (***B***), 2 m/s (***D***), and mTBI (***F***) animals. The two ROIs from the mTBI animals indicate the propagation of the hemodynamic response. Representative electrophysiological recordings of the extracellular field potential, high-frequency activity, and the total power (V^2^) of the high frequency from a sham (***G***) and mTBI animal (***H***). Cumulative data of the propagation rate (***I***), the SD extracellular field potential DC shift peak amplitude (***J***) and duration (***K***), and the duration of high-frequency suppression (***L***). Ketamine (120 mg/kg) was given systemically 30-min before impact and the presence of an SD was assessed using LSCI (***M***).

Histologic and immunohistochemical analyses were used to determine whether our mTBI model resulted in tissue damage and/or astrocyte activation. Previous studies have shown pathologic damage 24 h post-mTBI (McAteer et al., 2016; Tagge et al., 2018). Therefore, we sacrificed and perfused sham and mTBI animals 24 h post-treatment. To assess for gross tissue damage, we used Nissl staining to label the rough endoplasmic reticulum of all cells. We were unable to detect any significant gross tissue damage directly beneath the impactor (striatal section) or in more caudal sections (hippocampal sections) located on the perimeter of the impact zone in either the 2 m/s (sham *n* = 8; impacted *n* = 8; Fig. 3*A*) or 4 m/s (sham *n* = 8, mTBI *n* = 8; Fig. 3*B*) animals. We also used Prussian Blue staining to assess microbleeds and TUNEL staining to identify possible activated cell death. We used three to four sections that were physically separated (rostral to caudal) by ∼1–2 mm to span the entire brain. Microbleeds were very rare, with only two microbleeds in two separate animals observed. No microbleeds were observed in the other 18 sections from the remaining six mTBI animals, or from sham-treated animals (Fig. 3*C*). Likewise, TUNEL-positive neurons were extremely rare, with significant cell death being observed in only one of our mTBI animals (Fig. 3*D*). To more broadly test for cell death, we used FluoroJade-C staining in sham and mTBI (4 m/s) animals. We were unable to find a significant difference in the FluoroJade fluorescence between sham and mTBI animals (*t*_(16)_ = 0.812, *p* = 0.428; Fig. 3*E*,*G*; sham *n* = 8; mTBI *n* = 8). However, it is worth noting that the animal that did show positive TUNEL staining did have higher fluorescence in the FluoroJade-C stain. Another common feature of injury is neuroinflammation denoted by an increase in the expression levels of GFAP in astrocytes (Jones and Jarvis, 2017; Mountney et al., 2017). To assess this in our model we stained for GFAP 24 h post-mTBI. Representative images are shown in Figure 3*F*. We normalized the fluorescence intensity within the medial corpus collosum to that of the lateral somatosensory cortex in sham and mTBI animals. Again, we were unable to detect a difference in GFAP fluorescence (*t*_(16)_ = 1.295, *p* = 0.214; sham *n* = 8; mTBI *n* = 8; Fig. 3*H*). Due to the lack of histologic evidence of significant injury in the mTBI (i.e., 4 m/s) animals we did not extensively investigate damage in the 2-m/s impacted animals.

### mTBI impacts initiate SDs

To investigate the presence of SDs in mTBIs, we used LSCI and electrophysiology to monitor the CBF and the electrophysiological characteristics of SDs. A skin flap was generated to allow for LSCI but was placed over the skull during the impact. The skin flap did not negatively influence the impact, and the head deflected away from the impact, similar to impacts observed with the skin intact.

To monitor for hemodynamic responses associated with SDs we used LSCI. Following the sham or impact treatment the animal was immediately placed beneath the LSCI system within ∼3–5 s. In sham-treated animals (*n* = 11) the CBF remained consistent throughout the cortical surface during the 5 min of imaging (Fig. 4*A*,*B*). However, animals that received a 4 m/s impact displayed a propagating wave of hypoperfusion that slowly spread throughout the cortical surface. Representative images and corresponding ROIs are shown in Figure 4*C*,*D* (mTBI *n* = 12). The propagating waves of hypoperfusion have long been associated with SDs in mice (Tschirgi et al., 1957; Hansen et al., 1980; Paschen, 1984). The propagating wave of hypoperfusion (Phase I) was quickly followed by a brief increase in CBF (Phase II) that was then followed by a further reduction of CBF that resulted in longer term post-SD oligemia (Phase III). All of these phases are highlighted in the CBF trace (Fig. 4*D*). We further tested whether the slower impacts at 2 m/s produced similar propagating waves of hypoperfusion that are associated with SDs. Animals that were impacted at 2 m/s had a stable CBF with no indications of a propagating wave (2 m/s *n* = 4; Fig. 4*E*,*F*). Using the LSCI data we were able to calculate the propagation rate of the SDs by defining the wave front position over time. The impact induced SDs on average propagated at 3.5 mm/min (Fig. 4*M*), which is well within the published range of 2–6 mm/min across tissue and species (Gorelova and Bures, 1983; Guedes and Barreto, 1992; Martin et al., 1994; Aitken et al., 1998; Oliveira-Ferreira et al., 2010; Santos et al., 2016).

To confirm the presence of a SD we used electrophysiological recordings in sham and mTBI animals. Immediately following an impact, a glass electrode was inserted through a burr hole located over the visual cortex within 5 s of the impact (burr hole location shown in Fig. 1*B*). LSCI was simultaneously used to confirm that the electrode placement did not generate a SD. We were able to directly record the extracellular field potential shift and the suppression of high-frequency cortical activity, both of which are considered to be the gold-standard identifiers of SDs (mTBI *n* = 6; Fig. 4*H*). In sham-treated animals, we did not detect any shift in extracellular potential or suppression of high-frequency activity (Fig. 4*G*). The average peak amplitude of the SDs was 12.97 mV (Fig. 4*I*) with a duration of 1.06 min (Fig. 4*J*). These data are consistent with previously published properties of SDs evoked by other methods (i.e., KCl, application, or electrical stimulation) in C57/B6 mice (Takano et al., 2007; von Baumgarten et al., 2008; Lindquist and Shuttleworth, 2014, 2017; Enger et al., 2015; Ebine et al., 2016; Chen et al., 2017; Kucharz and Lauritzen, 2018). To measure the duration of suppression of the high-frequency activity, we plotted the total power (V^2^) of the high-frequency activity (Fig. 4*G*,*H*; Dreier et al., 2017). Recovery of the high frequency was considered to be the first spike in the total power above background (Dreier et al., 2017). The impact-induced SD resulted in an average of 6.88 min of high-frequency suppression (Fig. 4*K*).

Overall, we impacted 22 animals for these studies; 12 animals in our LSCI experiments (eight animals for the reperfusion and four animals for the multi-day recovery) and 10 animals in our electrophysiological studies. Of the 22 animals, SDs were detected in 18 animals (∼82%). Interestingly, the four animals that did not display a SD were all in the electrophysiology group. We did not monitor for SDs during the burr hole surgery required for electrophysiological recordings, and it is possible that a SD or other damage was introduced before the impact and impaired the ability to generate an SD with the impact. In the 18 animals that did have a confirmed SD, 16 of the 18 animals (89%) had a propagating SD in both hemispheres. The remaining two animals had a SD in one hemisphere only (one animal had a SD in the left hemisphere and the other in the right hemisphere). In these cases, the hemisphere that had an SD was used for subsequent analysis.

SD propagation is known to be sensitive to the non-selective NMDA receptor antagonist ketamine (Hernándéz-Cáceres et al., 1987; Kaube and Goadsby, 1994; Carlson et al., 2019). Ketamine was given systemically at least 30 min before the impact. Animals that were given saline alone displayed an impact-induced SDs in all seven animals (Fig. 4*M*). However, ketamine reduced the incidence of the impact-induced SDs (only two out of eight animals had an SD). These data suggest that the impact-induced SDs propagate via similar mechanisms to the SDs described in more severe injuries and in other models (Rashidy-Pour et al., 1995; Sakowitz et al., 2009; Hertle et al., 2012; Reinhart and Shuttleworth, 2018; Carlson et al., 2019).

### Impact-induced SDs produce prolonged deficits in CBF

**Figure 5.**
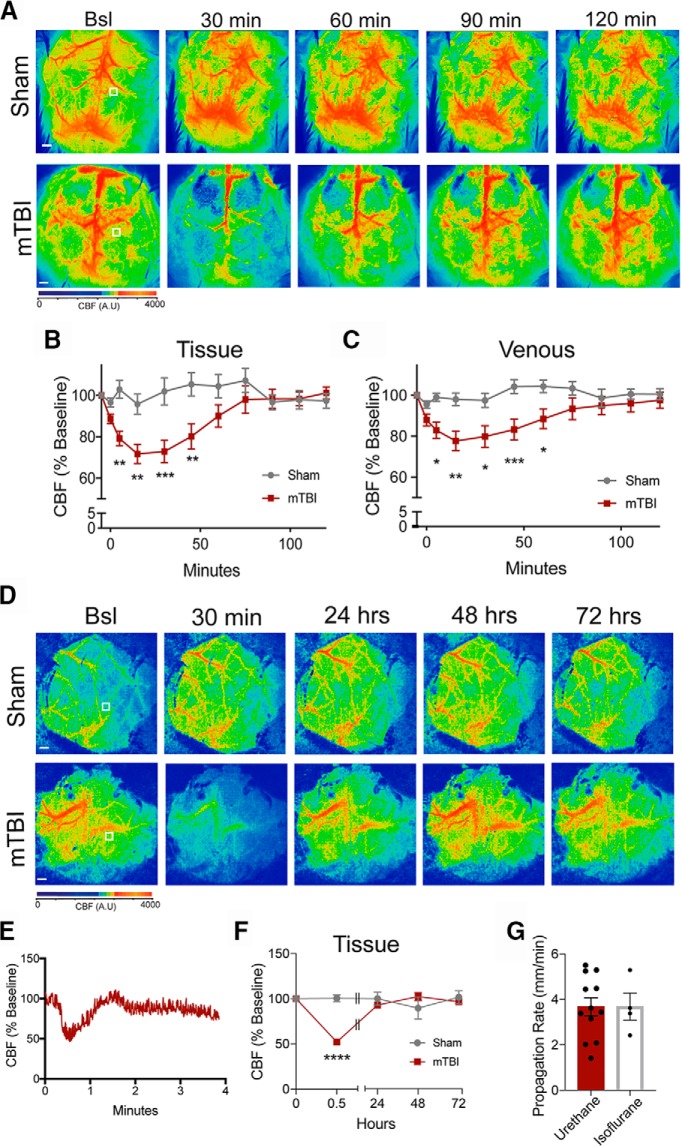
Impact-induced SDs are associated with long-term oligemia. Representative LSCI images of CBF from sham and mTBI animals (***A***). Representative ROIs indicate the location of repeated measures of CBF in the tissue. CBF was quantified over the 120-min period and plotted over time for sham and mTBI animals in both tissue (***B***) and venous (***C***) regions. Modified cranial windows were generated to allow for repeated measures of CBF immediately after the impact and for subsequent days. Representative images before the impact, 30 min post-impact, and subsequent days are shown (***D***). Representative ROIs indicate where the CBF was quantified. Animals were anesthetized with isoflurane rather than urethane. Representative trace of the hemodynamic responses that are associated with the propagating SD in the presence of isoflurane anesthesia (***E***). CBF was quantified and normalized to pre-impact baseline. Using LSCI were able to confirm the SD and the peak reduction of CBF and subsequent days following the impact (***F***). The propagation rate was also quantified in the presence of isoflurane anesthesia (***G***). Scale bars = 500 μm.

CBF responses to SDs are complex, and can be dependent on species and metabolic status before the SD (for review, see Ayata and Lauritzen, 2015). We used LSCI to investigate the long-term CBF changes after an impact-induced SDs. The CBF in sham-treated animals remained relatively stable throughout the 120-min recording period (Fig. 5*A*). Baseline LSCI images were taken before impact and SDs were confirmed by the propagating wave of hypoperfusion (Fig. 4*E*). Images were then acquired every 15 min to monitor CBF recovery. Representative images are shown in Figure 5*A*. CBF was reduced ∼20–30% at 30 min and did not fully recover to baseline levels until ∼90 min post-impact in both venous and tissue regions (sham *n* = 8; mTBI *n* = 8; Fig. 5*B*,*C*; Table 1). The venous blood flow dropped to ∼20% of the baseline levels compared to the 30% reduction in the tissue flow, but recovered in a similar time course. The time course of the CBF recovery shown here is similar to what has been previously reported from SDs initiated by other means (von Baumgarten et al., 2008; Piilgaard and Lauritzen, 2009; Leung et al., 2013) and other closed skull impact models by Bouley and colleagues (Bouley et al., 2019).

In another cohort of animals, we investigated the stability of CBF recovery by monitoring the CBF every 24 h for 3 d. Impact-induced SDs were confirmed immediately after the mTBI and the degree of oligemia was determined at 30 min post-mTBI. For these experiments we used isoflurane as the anesthetic due to the speed of onset and recovery. Isoflurane is known to reduce the frequency of SDs (Takagaki et al., 2014; Balança et al., 2017). However, we detected a SD following each impact in all four animals. A representative trace is shown in Figure 5*E*. Isoflurane did not significantly alter the hemodynamic response (Fig. 5*E*) or propagation rate of the SDs (Fig. 5*G*). However, the CBF at 30 min was significantly lower in the mTBI animals compared to the sham-treated animals (*F*_(4,40)_ = 13.38, *p* < 0.0001; Fig. 5*F*). Interestingly, the CBF was slightly lower in the isoflurane-treated animals relative to the urethane-treated animals at 30 min post-mTBI (∼70% in urethane vs ∼50% in isoflurane). In the mTBI animals the CBF at 24, 48, and 72 h was similar to pre-mTBI levels (Fig. 5*D*,*F*).

## Discussion

Our studies are the first to directly record the electrophysiological properties of SDs in a closed skull model of mTBIs and to correlate the presence of a SD with mTBI-like behavior. The mTBI-like behavior was not long-lasting and did not result in significant long-term behavioral or cognitive deficits. The injury did not produce gross tissue damage, cell death, or astrocyte activation. The impact-induced SDs were associated with long-term reductions in CBF. Interestingly, slower impacts did not result in SDs and were not associated mTBI-like behavior. Overall our data suggest that SDs may play a critical role in mTBIs.

Our data suggest that immediately following impact, the mTBI animals took longer to regain movement and had fewer episodes of movement in a 10-min trial. However, this reduced mobility is not long-lasting. There were no significant differences between the movement of sham or mTBI animals in the following hours and days. This is similar to previous studies using closed skull impacts (Shen et al., 2011). Shen and colleagues showed no difference in the total distance traveled or time of movement in the hours following a closed skull weight drop model (Shen et al., 2011). However, McAteer and colleagues found that animals that had a single closed skull injury displayed fewer traveled squares six weeks post-injury (McAteer et al., 2016). This discrepancy may be due to species differences (rat vs mouse), but the injuries were similar. Behavioral and cognitive deficits are common in more severe penetrating type injuries. However, the behavioral and cognitive deficits are subtler in closed skull injuries. Using a similar closed skull impactor model, Prins and colleagues found significant deficits the novel object recognition task 24 h post-injury (Prins et al., 2013). However, we were unable to detect a deficit in our paradigm. This may be due to the difference in delay period before the novel object presentation. Prins and colleagues used a 24-h delay for their study and ours was only 5 min. The 24-h delay requires memory consolidation and recall. With a similar mTBI model, Marschner and colleagues found mTBI animals had a stronger freezing response in the contextual fear paradigm (Marschner et al., 2019). Again, we were unable to detect a difference in our model. However, the timing of the fear-based task was slightly different between the two studies (3 d vs 24 h for our study). The exacerbated fear response that they found was resolved by five weeks post-mTBI. Similar to the novel object task, the increased delay between testing periods increases the difficulty of the task. Gait abnormalities are much more common in more severe penetrating injuries, especially when the motor cortex is involved (Neumann et al., 2009; Mulherkar et al., 2017; Ouyang et al., 2017; Pöttker et al., 2017; Baker et al., 2019; Kinder et al., 2019). Using a slightly more aggressive closed skull model, Mountney and colleagues could detect slight abnormalities in gait using the CatWalk analysis system, but we were unable to detect differences in our model (Mountney et al., 2017). Overall, mTBIs can have a myriad of deficits in behavior, cognition, and learning and memory, but it is clear that the deficits are graded on the severity of the injury.

SDs have long been associated with moderate and severe TBIs in rodents and more recently in humans (Hermann et al., 1999; Strong et al., 2002; Rogatsky et al., 2003; Fabricius et al., 2006; Hartings et al., 2008, 2009, 2011; von Baumgarten et al., 2008; Sakowitz et al., 2009; Leung et al., 2013; Sword et al., 2013; Balança et al., 2017; Hosseini-Zare et al., 2017; Carlson et al., 2019). Some rodent models of mTBIs have shown the presence of SDs. However, prior studies have used more invasive techniques which often result in significant tissue swelling and/or bleeding, both of which can cause SDs (Sunami and Nakamura, 1989; Nilsson et al., 1993; Rogatsky et al., 2003; Sword et al., 2013). Recent data from Bouley and colleagues have shown propagating waves of hypoperfusion that are associated with SDs in a closed skull weight drop model (Bouley et al., 2019). In that study they used a unilateral weight drop model that consisted of a 50-g weight being dropped from a height of 15 cm (Bouley et al., 2019). This means that at the site of impact the tip was traveling at ∼1.72 m/s. This is significantly slower than our model, but the experimental setup was much different. In their study the impact was centered over one hemisphere and it is unclear if the head was restricted. Our model seems to be less severe in that we did not detect significant neurodegeneration or seizures. In our hands, we detected SDs in 18 of the 22 animals that were impacted (82%). The incidence of SDs in the weight drop model published by Bouley and colleagues was closer to 60% (Bouley et al., 2019). Nonetheless, in both cases the presence of SDs was associated with concussion-like behavior. Bouley and colleagues showed increased neuronal cell death and microbleeds in the injured hemisphere. In our model, we were able to detect a single microbleed in two of our eight mTBI animals and TUNEL-positive nuclei in only one of mTBI animals. This is significantly fewer than what is reported by Bouley and colleagues. This suggests that our model is less severe than that of the weight drop model, but somewhat more efficient in generating SDs. This difference may be due to the surface area of the injury. Our studies use a larger 5-mm diameter tip that distributes the force over both hemispheres, whereas Bouley and colleagues used a 2-mm Delrin tip to transmit the weight drop energy to a single hemisphere. Another difference between the studies is when the histology was done. Bouley and others looked for apoptotic cells 48 h post-injury, whereas ours were done 24 h post-injury.

Our studies indicate that the impact-induced SDs are sensitive to ketamine inhibition. This demonstrates that the SDs generated in our model propagate via similar mechanisms to SDs in more severe injuries when there is tissue damage and bleeding. This also provides a critical starting point for the development of pharmacological interventions that target SDs directly. One caveat within our experimental design is that the behavioral data were gathered using isoflurane to allow for rapid recovery and detection of acute behavioral that may be due to the SD event itself. Isoflurane is known to reduce the frequency of SDs (Takagaki et al., 2014; Balança et al., 2017), and may confound our behavioral data. However, our extended CBF measurements across days did use isoflurane and all four impacts induced an SD. Furthermore, isoflurane did not alter the hemodynamic response or propagation rate. However, isoflurane did affect the peak reduction of CBF at 30 min post-impact (50% in isoflurane vs 30% in urethane).

In the original manuscript describing SDs, Leao noted that these events could be initiated with a light touch to the cortical surface with a glass rod (Leao, 1944). *Ex vivo* and *in vitro* studies have supported the idea of mechanical compression and/or stretch of neuronal tissue as one possible initiator of SDs (Geddes-Klein et al., 2006; Cater et al., 2007). There is likely a critical volume of brain tissue that must depolarize in order for SD initiation (Matsura and Bures, 1971; Tang et al., 2014). In our studies, the rapid acceleration of the head could result in the compression of the entire cortex against the skull. Our LSCI data showing that SDs originate near the impact site imply a focal region of activation sufficient to reach threshold for SD initiation under the impact site. The direct mechanism that links cortical compression to neuronal depolarization is still unresolved. However, the presence of mechanical transducing ion channels is an intriguing possibility (Sachs, 2015). Ischemia itself is thought to be a critical initiator of SDs in different injury models, and is well supported by *in vivo* (Hartings et al., 2003; Oliveira-Ferreira et al., 2010, 2019; von Bornstädt et al., 2015) and *ex vivo* (Luhmann and Kral, 1997; Aitken et al., 1998; Andrew, 2005; Takano et al., 2007) reports. Bouley and colleagues showed that the presence of a SD was associated with increased numbers of microbleeds underneath the injury site in their concussion model (Bouley et al., 2019). The microbleeds could be a potential initiator of the SDs in that model, although this remains to be directly tested. In our studies, microbleeds were very rare, and SDs were usually generated in cortex without detectable blood brain barrier disruption.

Our data indicate that the impact-induced SDs are associated with a prolonged reduction in CBF that recover within 90 min. This recovery remains relatively stable in the subsequent days. The peak reduction and recovery phase in our studies are very similar to those presented by Bouley and colleagues (Bouley et al., 2019). The link between long-term reduction in CBF (oligemia) and SDs has been well established in mice. However, the hemodynamic responses associated with SDs can vary widely within a given species and especially across species. In higher order animals and in humans, SDs most often trigger a propagating wave of increased CBF (hyperemia). However, both hemodynamic response can result in long-term post-SD oligemia (Hinzman et al., 2014; Ayata and Lauritzen, 2015). During this period of post-SD oligemia, there is an uncoupling between the vascular supply and the neuronal demand (Takano et al., 2007; Piilgaard and Lauritzen, 2009; Piilgaard et al., 2011; Ayata, 2013; Koide et al., 2013; Hinzman et al., 2014; Ayata and Lauritzen, 2015; Toth et al., 2016). In the wake of an SD there is a period of persistent vasoconstriction due to elevated levels of extracellular K^+^ and a decrease in nitric oxide (Ayata and Lauritzen, 2015). Altered CBF has long been associated with concussions and can be a useful readout for long-term recovery (Len and Neary, 2011; Giza and Hovda, 2014; Keightley et al., 2014; Sours et al., 2015; Barlow et al., 2017). The use of CBF as a readout for mTBIs has been limited due to the variability between individuals. Another limitation of using CBF as a diagnostic tool has traditionally been the cost and access to MRI-based imaging in a time frame that is relevant to the acute mechanisms described in the present study. However, advancement of near-infrared spectroscopy (NIRS) approaches could provide some significant advantages, and has already shown some promise as a diagnostic tool for concussions (Urban et al., 2015; Bishop and Neary, 2018; Forcione et al., 2018).

Overall, our data demonstrate the presence of SDs in mTBIs and suggest that SDs may play a role in concussion-like behavior. These data provide significant insight into the cellular and physiologic mechanism that may underlie concussions.

## Synthesis

Reviewing Editor: Jun Chen, University of Pittsburgh Decisions are customarily a result of the Reviewing Editor and the peer reviewers coming together and discussing their recommendations until a consensus is reached. When revisions are invited, a fact-based synthesis statement explaining their decision and outlining what is needed to prepare a revision will be listed below. The following reviewer(s) agreed to reveal their identity: Baptiste Balanca, Jed Hartings.

Both reviewers still have many relatively minor concerns. Therefore, additional revisions are required before a final decision can be made.

Reviewer #1

This manuscript entitled “Spreading depolarizations occur in mild traumatic brain injuries and are associated with post-injury behavior” investigates electrophysiological, regional cerebral blood flow (rCBF), histological and behavioral changes early after mild TBI (mTBI) in young mice. The authors report that the SD induced by mTBI is responsible for rCBF reduction and neuronal activity transient interruption. They suggest that this observation underlie the acute behavioral changes observed after mTBI that recovers after 1-2h, as did neuronal activity and rCBF levels.

In addition to methodological and discussion improvement, the authors provided further results that improve the relevance of the herein manuscript.

The behavioral monitoring has been described in detail, namely what happened in the following hours and days after the impact. The complete recovery of the animal without observable histological lesions is an additional argument in support of the accountability of spreading depolarization in animals transient motionless after the impact.

The authors used urethane for the acute speckle and electrophysiological recording and isoflurane for other experiments. They have provided informative results about the absence of anesthetic significant effect on SD occurrence, propagation and consequences on rCBF changes following SD induced by mTBI. The corresponding section in the discussion is also informative.

The effect of ketamine that reduced SD incidence after mTBI show that the reported depolarization fulfilled the traditional characteristics of spreading depolarization. It would have been relevant to know if ketamine also reduces the number of motionless animal following mTBI.

Methodological details about electrophysiological and imaging details that have been discussed during the previous review have also been filled in, as well as the figure heatmaps and scales.

There are still some minor typing and references mistakes:

- line 35 injurea instead of injuries

- ref Brent et al : page 108 missing

- ref Matsuura et al : page 238-49 missing

Reviewer #2

This revised manuscript adequately addresses the concerns raised in the previous review. The paper contributes new information about the behavioral consequences of mTBI, likely related to spreading depolarization (SD) pathophysiology, and their time course over minutes, hours, and days. To their credit, the authors have added an extensive battery of behavioral testing that convincingly demonstrate the the neurologic deficits are transient, and thus mimic the symptomology of most concussion victims. The link of neurologic deficits to a mechanism (SD) in concussion is novel and advances the field. However, the paper could should still be improved in a few areas.

The writing of the manuscript would be improved by the assistance of a medical editor. There are multiple cases of incorrect words and improper sentence construction. For instance:

In the abstract, “...and are sensitive to pharmacological inhibition by ketamine” does not have a proper subject.

Line 35: injuries

Line 54: studied extensively studied

Line 270: “mTBI animals on took”

Lines 283-284 and 392-394 are not sentences.

Lines 326-328: Was it intended to write that “we were NOT able to find”? And “between sham and mTBI” would be better than “within the cortex of both sham and mTBI”

Line 391: “impacted induced” and “animlas”

Line 406: use of ‘relative’ is confusing when discussing CBF, which is a relative measure. Would ‘compare’ be better?

Line 424-425:

Line 431: data (plural) suggests (singular)

Line 432: episodes of movement

Line 463: more invasive “techniques”

Line 471: “however” should not be used as a conjunction

Line 475: 14 of the 16 animals

Line 490: incidents should be incidence

Line 409-410: reductions (plural) in CBF that recovers (singular)

Throughout the section “mTBI impacts initiate spreading depolarizations”, the references to figures 3 and 4 are often confused and inaccurate. They should all be reviewed and corrected. In the same section, the animal numbers are still confusing. They report 4 sham, 8 mTBI 4m/s, and 4 mTBI 2 m/s animals. As written, it's reported that the 4 m/s animals all had SDs, and the others didn't. It's then stated however, that in total 16 out of 20 animals had SDs. This doesn't add up. In the next paragraph a further 8 animals are reported. They also discuss ‘variability’ in whether SDs occur, and what may cause it, which adds to the confusion since it was reported that all 4 m/s animals had SDs and no others did. Doesn't variability only exist between groups, which is explained by the faster impact of 4 m/s? This all needs to be clarified.

The authors should also comment on whether microbleeds were present, in light of the results of Bouley et al. This has primary relevance to the discussion of what causes the SD, as some contend that SD does not occur without ischemia, caused in this case by possible microbleeds. Prussian blue staining would add considerably to the impact of the paper, as would TUNEL staining to rule out cell death more completely. Proving the negative is not possible, but a claim of no cellular pathology in this model is likely to be controversial.

## Author Response

Dear Dr. Chen

We are very excited by the opportunity to publish our manuscript in eNeuro. Below you will find our responses to all the reviewers' comments in red. All the changes within the manuscript are highlighted in red font as well. Thank you again for this opportunity.

Russell A. Morton

Assistant Professor

Department of Neurosciences

University of New Mexico Health Science Center

Albuquerque, NM 87131

Ph. 505-272-8815

---------------------------------------------

Synthesis of Reviews:

Computational Neuroscience Model Code Accessibility Comments for Author (Required):

N/A

Significance Statement Comments for Author (Required):

The interesting data reported in the current manuscript confirm that any brain injury can lead to an SD even if there is no histological damage. Moreover, this suggest that the transient neurological deficit (e.g. loss of consciousness or confusion) could be due to the occurrence of SDs triggered by the injury.

Comments on the Visual Abstract for Author (Required):

N/A

Synthesis Statement for Author (Required):

Both reviewers still have many relatively minor concerns. Therefore, additional revisions are required before a final decision can be made.

Reviewer #1

This manuscript entitled “Spreading depolarizations occur in mild traumatic brain injuries and are associated with post-injury behavior” investigates electrophysiological, regional cerebral blood flow (rCBF), histological and behavioral changes early after mild TBI (mTBI) in young mice. The authors report that the SD induced by mTBI is responsible for rCBF reduction and neuronal activity transient interruption. They suggest that this observation underlie the acute behavioral changes observed after mTBI that recovers after 1-2h, as did neuronal activity and rCBF levels.

In addition to methodological and discussion improvement, the authors provided further results that improve the relevance of the herein manuscript.

The behavioral monitoring has been described in detail, namely what happened in the following hours and days after the impact. The complete recovery of the animal without observable histological lesions is an additional argument in support of the accountability of spreading depolarization in animals transient motionless after the impact.

The authors used urethane for the acute speckle and electrophysiological recording and isoflurane for other experiments. They have provided informative results about the absence of anesthetic significant effect on SD occurrence, propagation and consequences on rCBF changes following SD induced by mTBI. The corresponding section in the discussion is also informative.

The effect of ketamine that reduced SD incidence after mTBI show that the reported depolarization fulfilled the traditional characteristics of spreading depolarization. It would have been relevant to know if ketamine also reduces the number of motionless animal following mTBI.

We completely agree with the reviewer. However, the doses of ketamine that are required to block SDs also have anesthetic properties. This limits our ability to use this drug during behavioral assessment. Current studies are investigating other drugs that target the pannexin 1 and/or adenosine receptors that do not have anesthetic effects that could be utilized in future studies.

Methodological details about electrophysiological and imaging details that have been discussed during the previous review have also been filled in, as well as the figure heatmaps and scales.

There are still some minor typing and references mistakes:

- line 35 injurea instead of injuries

- ref Brent et al : page 108 missing

- ref Matsuura et al : page 238-49 missing

All of the above mistakes have been corrected and are highlighted with red font.

Reviewer #2

This revised manuscript adequately addresses the concerns raised in the previous review. The paper contributes new information about the behavioral consequences of mTBI, likely related to spreading depolarization (SD) pathophysiology, and their time course over minutes, hours, and days. To their credit, the authors have added an extensive battery of behavioral testing that convincingly demonstrate the the neurologic deficits are transient, and thus mimic the symptomology of most concussion victims. The link of neurologic deficits to a mechanism (SD) in concussion is novel and advances the field. However, the paper could should still be improved in a few areas.

The writing of the manuscript would be improved by the assistance of a medical editor. There are multiple cases of incorrect words and improper sentence construction. For instance:

In the abstract, “...and are sensitive to pharmacological inhibition by ketamine” does not have a proper subject.

Line 35: injuries

Line 54: studied extensively studied

Line 270: “mTBI animals on took”

Lines 283-284 and 392-394 are not sentences.

Lines 326-328: Was it intended to write that “we were NOT able to find”? And “between sham and mTBI” would be better than “within the cortex of both sham and mTBI”

Line 391: “impacted induced” and “animlas”

Line 406: use of ‘relative’ is confusing when discussing CBF, which is a relative measure. Would ‘compare’ be better?

Line 424-425:

Line 431: data (plural) suggests (singular)

Line 432: episodes of movement

Line 463: more invasive “techniques”

Line 471: “however” should not be used as a conjunction

Line 475: 14 of the 16 animals

Line 490: incidents should be incidence

Line 409-410: reductions (plural) in CBF that recovers (singular)

All of the above edits were corrected and highlighted in red. We have also gone through the manuscript and made necessary edits to increase clarity.

Throughout the section “mTBI impacts initiate spreading depolarizations”, the references to figures 3 and 4 are often confused and inaccurate. They should all be reviewed and corrected. In the same section, the animal numbers are still confusing. They report 4 sham, 8 mTBI 4m/s, and 4 mTBI 2 m/s animals. As written, it's reported that the 4 m/s animals all had SDs, and the others didn't. It's then stated however, that in total 16 out of 20 animals had SDs. This doesn't add up. In the next paragraph a further 8 animals are reported. They also discuss ‘variability’ in whether SDs occur, and what may cause it, which adds to the confusion since it was reported that all 4 m/s animals had SDs and no others did. Doesn't variability only exist between groups, which is explained by the faster impact of 4 m/s? This all needs to be clarified.

We thank the reviewer for their thorough reading of our manuscript. We have edited the section mentioned and have corrected the figure references. We have also added a section describing the overall numbers of animals and what experiments they were used for. We hope that this clarifies the animal numbers.

The authors should also comment on whether microbleeds were present, in light of the results of Bouley et al. This has primary relevance to the discussion of what causes the SD, as some contend that SD does not occur without ischemia, caused in this case by possible microbleeds. Prussian blue staining would add considerably to the impact of the paper, as would TUNEL staining to rule out cell death more completely. Proving the negative is not possible, but a claim of no cellular pathology in this model is likely to be controversial.

We agree with the reviewer that bleeding in the brain is critical component to injury and the initiation of SDs. To address this, we went back and stained a subset of our tissue sections with Prussia Blue to assess for micro-bleeds. We stained 3-4 section per brain. We did detect a single microbleed in 2 separate mTBI animals and none in the slices from the remaining 6 mTBI animals or sham treated animals. This was a fairly rare event. We also stained 3-4 sections per brain with the TUNEL stain to rule out cell death. We did detect TUNEL positive nuclei from one of our mTBI animals. We have added all of these findings to the manuscript.
